# Surgical Treatment of Obstructive Sleep Apnea Syndrome in Patients with Bilateral Vocal Fold Paralysis

**DOI:** 10.3390/jcm15145373

**Published:** 2026-07-09

**Authors:** Magdalena Marków, Agata Sybila, Paweł Ścierski, Monika Kozyra, Marzena Dwojak-Szafruga, Maciej Misiołek, Wojciech Ścierski

**Affiliations:** 1 Department of Otorhinolaryngology and Oncological Laryngology, Faculty of Medical Sciences in Zabrze, Medical University of Silesia in Katowice, Marii Curie-Sklodowskiej 10, 41-800 Zabrze, Poland; 2 Department of Internal Medicine, Municipal Hospital in Tychy, Cicha 27, 43-100 Tychy, Poland

**Keywords:** bilateral vocal fold paralysis, obstructive sleep apnea, OSA, polysomnography

## Abstract

**Background**: Systematic among patients with bilateral vocal fold paralysis (BVFP), the most clinically significant symptom is dyspnea, which often requires surgical intervention. One of the most common causes of BVFP is iatrogenic injury during thyroidectomy. Some patients report not only day time breathing difficulties but also a deterioration in sleep quality since the onset of BVFP. Obstructive sleep apnea (OSA) is a common sleep-related breathing disorder caused by recurrent upper airway obstruction during sleep. Although it is usually associated with pharyngeal collapse, fixed laryngeal obstruction has also been suggested as a potential contributor to sleep-disordered breathing. Therefore, we aimed to assess the impact of surgical treatment of OSA in patients with BVFP. **Methods**: Between 2022 and 2025, 18 patients with BVFP were screened. Patients who met diagnostic criteria for OSA based on preoperative polysomnography (PSG) and the Epworth Sleepiness Scale (ESS) AHI ≥ 15 events/h or AHI ≥ 5 events/h with associated symptoms were included in the study. Five female patients met the criteria and underwent arytenoidectomy combined with posterior cordectomy. A follow-up PSG and re-evaluation of ESS were performed 6–12 months after surgery, and patients were asked about their subjective perception of sleep quality improvement. **Results**: An improvement in sleep quality, decrease in the median ESS score and respiratory parameters showed an improvement, although this was not statistically significant due to the small sample size (n = 5). **Conclusions**: These results indicate a positive postoperative trend in sleep quality among patients with BVFP however, given the limited sample size, further studies are required to confirm these observations.

## 1. Introduction

Bilateral vocal fold paralysis (BVFP) is characterized by the immobilization of the vocal folds in a medial or, less commonly, intermediate position due to injury of the recurrent laryngeal nerves (RLNs) [1,2]. Iatrogenic injury is one of the most frequently reported causes of BVFP, accounting for approximately 26–59% of cases, most commonly following surgical procedures in the neck and thoracic region. Thyroid surgery represents the leading cause, responsible for approximately one quarter of all cases of BFVP [3]. The most severe clinical manifestation is laryngeal dyspnea, which in advanced cases may be life-threatening and require immediate airway intervention, such as endotracheal intubation or tracheotomy [2]. A systematic review analyzing 55 studies estimated that approximately 36% of patients with BVFP require tracheotomy [1]. Despite its high effectiveness in securing airway patency, this procedure is currently regarded as the least preferred option because of the requirement for long-term tracheostomy care and the substantial psychosocial burden imposed on patients [2].

If BVFP occurred from iatrogenic nerve injury, a period of observation lasting 6–12 months is generally recommended to allow for potential reinnervation and spontaneous recovery of RLN function. After this interval, if symptoms persist or worsen, surgical intervention is indicated. The most commonly employed surgical treatments for BVFP include arytenoidectomy, vocal fold lateralization (laterofixation) and posteriorcordotomy, all aimed at improving airway patency and relieving respiratory symptoms [4].

Obstructive sleep apnea (OSA) is one of the most common sleep-related breathing disorders and is characterized by recurrent episodes of partial or complete upper airway obstruction during sleep. These events result in intermittent hypoxemia, recurrent arousals, and disruption of normal sleep architecture. According to a systematic review, OSA affects approximately 13–33% of men and 6–19% of women in the general adult population. Despite its high prevalence, OSA remains substantially underdiagnosed, with global estimates suggesting that nearly one billion adults are affected worldwide. Beyond its impact on sleep quality, OSA is recognized as an important risk factor for cardiovascular and metabolic diseases, neurocognitive dysfunction, and impaired daytime functioning [5].

Although OSA most commonly results from dynamic collapse of the pharyngeal airway, fixed upper airway obstruction at the level of the larynx may also contribute to sleep-disordered breathing [6,7,8,9]. In patients with BVFP, persistent narrowing of the glottic air-way results from the inability of the paralyzed vocal folds to abduct during inspiration, creating a fixed upper airway obstruction. This narrowing increases inspiratory air flow resistance and velocity, generating negative pressure gradients in the upper airway. During sleep, when pharyngeal dilator muscle activity is physiologically reduced, these negative pressure swings promote collapse of the compliant pharyngeal structures located above the level of the glottis. Consequently, although the obstruction in BVFP is located at the laryngeal level, it may facilitate secondary pharyngeal collapse and contribute to the development or aggravation of OSA [7,10]. Despite this plausible pathophysiological relationship, the prevalence and clinical significance of OSA in patients with BVFP remain poorly characterized. Therefore, it remains unclear whether surgical enlargement of the glottic airway results in clinically meaningful improvement in OSA severity. Despite the limited sample size, the aim of the present study was to evaluate the effectiveness of surgical treatment for BVFP in patients with confirmed OSA and to assess its impact on sleep-related respiratory outcomes.

## 2. Materials and Methods

The study was conducted in accordance with the Declaration of Helsinki and approved by the Ethics Committee of Medical University of Silesia (Approval No. PCN/CBN/0052/KB1/3/22 approved on 22 February 2022). Written informed consent was obtained from all participants prior to enrollment.

Eighteen patients with BVFP were assessed for eligibility for inclusion in the study, which was conducted between 2022 and 2025. All patients underwent overnight polysomnography (PSG) as part of the diagnostic work-up. Patients with an apnea–hypopnea index (AHI) > 5 events/h were considered eligible for inclusion. Six patients met the inclusion criterion; however, one patient withdrew from the study after experiencing a stroke. Consequently, five female patients completed the study and were included in the final analysis.

None of them experienced dyspnea severe enough to require a tracheotomy. The median age was 62 years (IQR 54–64), and the median preoperative BMI was 29.7 kg/m^2^ (IQR 21.7–32.9). The arytenoidectomy combined with posterior cordectomy was performed using a Kleinsasser micro laryngeal surgery set (Karl Storz SE & Co. KG, Tuttlingen, Germany) under an operating microscope. A CO_2_ laser was used to excise the arytenoid cartilage along with approximately 1/3 of posterior vocal fold unilaterally. PSG was performed both preoperatively and 6–12 months post operatively using polysomnography equipment, and patients with an apnea–hypopnea index (AHI) > 5 were included. This threshold was applied because, according to the national diagnostic criteria used in the authors’ country, obstructive sleep apnea (OSA) is diagnosed in patients with an AHI ≥ 15 events/h regardless of symptoms, or with an AHI ≥ 5 events/h in the presence of characteristic clinical symptoms, such as excessive daytime sleepiness, loud snoring, or nocturnal dyspnea, which were reported by the included patients. Sleepiness was assessed using the Epworth Sleepiness Scale (ESS), and patients were asked whether they perceived an improvement in sleep quality following surgery (YES or NO answers). Due to the small sample size and non-normal distribution of the data, results are presented as median and interquartile range (IQR). Pre- and postoperative values were compared using the Wilcoxon signed-rank test.

## 3. Results

The results are presented in the Table 1. The study group consisted of five female patients. All patients reported a subjective improvement in sleep quality following surgery. A decrease in the median ESS score was observed, indicating an improvement in daytime sleepiness. Although postoperative reductions were observed in several respiratory parameters, none of these changes reached statistical significance due to the small sample size (n = 5).

The median AHI decreased from 17.1 (IQR 6.65–61.75) preoperatively to 7.8 (IQR 4.2–15.0) postoperatively (*p* = 0.125). Snoring time improved from 72.7% (IQR 47.8–73.85) to 44.8% (IQR 11.7–81.95; *p* = 0.625). The oxygen desaturation index (ODI) decreased from 39.5 (IQR 6.6–58.5) to 4.4 (IQR 1.5–12.1; *p* = 0.0625).

There was also an improvement in the number of apneas and hypopneas. Mean oxygen saturation and mean desaturation showed relatively minor changes, with slight improvement; the median mean saturation was 92.1% (IQR 91–95.3) preoperatively and 92.3% (IQR 91.05–95.6) postoperatively (*p* = 0.625), and the median time with SpO_2_ < 90% decreased from 5.3% (IQR 0.1–19.2) to 2.7% (IQR 0–9.4; *p* = 0.625).

## 4. Discussion

The most significant clinical manifestations in patients with BVFP have been reported to include exertional dyspnea, respiratory distress, and characteristic inspiratory stridor [1]. Less frequently, nocturnal symptoms such as snoring, fragmented sleep, and non-restorative sleep have also been described, potentially contributing to daytime fatigue and impaired overall quality of life [7,8]. However, the available scientific literature specifically addressing sleep quality and snoring in patients with BVFP remains limited.

In a study published in 2003 involving 14 patients with BVFP, unilateral arytenoidectomy was associated with a significant improvement in snoring parameters, including a reduction in the proportion of loud snoring episodes and an increase in the percentage of sleep without snoring. Specifically, loud snoring decreased from approximately 53.8 to 31.6 (*p* = 0.006), while the proportion of sleep without snoring increased from approximately 26.9 to 46.6 (*p* = 0.01) [6]. Similarly, a 2002 study including nine BVFP patients who underwent arytenoidectomy documented a decrease in the snoring index following surgical intervention [8].

In addition, a case report described a 48-year-old woman with previously undiagnosed BVFP of unknown etiology, diagnosed for OSA, in whom surgical vocal fold lateralization was followed by decrease of OSA symptoms [9]. It should be emphasized that comprehensive diagnostic evaluation is essential in patients with OSA, as underlying laryngeal disorders may contribute to persistent symptoms despite standard therapy. For instance, a case report described a 27-year-old patient with OSA diagnosed by PSG whose symptoms did not fully resolve despite continuous positive airway pressure (CPAP) therapy. Further evaluation, including endoscopic examination, revealed previously unrecognized idiopathic BVFP [7]. Another example is a case of a 46-year-old woman presenting with long-standing hoarseness and excessive daytime sleepiness. PSG revealed severe OSA with an AHI of 72/h. Treatment with CPAP was initiated but did not result in clinical improvement. Further examination revealed BVFP; it occurred that the patient had undergone thyroid surgery 30 years earlier. Endoscopic bilateral posterior cordotomy was performed, resulting in a reduction in AHI to 4.4/h and clinical improvement [11].

Another observation highlights that sleep-disordered breathing may also occur as a consequence of subacute or procedure-related vocal fold dysfunction. This is illustrated by a case report of a 55-year-old patient who developed symptoms consistent with mild obstructive sleep apnea after vocal fold augmentation. In this case, the symptoms were attributed to a hematoma at the injection site, and symptomatic treatment with CPAP resulted in clinical improvement until the hematoma resolved [12].

In the present study, surgical intervention for BVFP was associated with improvements in nocturnal respiratory parameters, including median oxygen desaturation index (ODI), the number of apneas, and hypopneas; however, these changes did not reach statistical significance, most likely due to the small sample size (n = 5). The small sample size of the study group results from the fact that a considerable proportion of patients with BVFP require tracheotomy due to severe dyspnea and the need to secure the airway. The presence of a tracheotomy prevents reliable assessment of sleep-disordered breathing, which excludes such patients from the analysis. Despite the lack of statistical significance, the observed reduction in median ODI from 39.5 to 4.4 (*p* ≈ 0.0625) may still be clinically relevant. Lower ODI values reflect fewer nocturnal oxygen desaturation episodes, which may reduce intermittent hypoxia and its adverse physiological consequences. Likewise, the observed decrease in AHI suggests a trend toward improved nocturnal respiratory stability. Nevertheless, given the limited statistical power of the present study, these observations should be considered preliminary.

It has been well established that recurrent hypoxic episodes and disturbances in sleep architecture promote chronic inflammation predisposing to the development of multiple chronic conditions, particularly affecting the cardiovascular, neurological, pulmonary, and endocrine systems. Among the most commonly reported comorbidities are arterial hypertension, diabetes mellitus, coronary artery disease, and ischemic heart disease [13].

A concurrent decrease in median ESS scores was observed, suggesting reduced daytime sleepiness, improved concentration, and enhanced overall daily functioning. Although mean oxygen saturation values remained relatively stable, the duration of desaturations below 90% was shortened, which may confer additional clinical benefit. Furthermore, the reduction in the number of apneas and hypopneas suggests improved nocturnal ventilation and respiratory stability. All patients reported improved sleep quality after surgery, underscoring the positive effect of the intervention on daily functioning. Despite the observed postoperative trends toward improved respiratory- and sleep-related parameters, the findings should be interpreted with caution. The small sample size substantially limited the statistical power of the study and precluded adjustment for potential confounding variables, including age and body mass index, as well as subgroup analyses. In addition, because the study population consisted exclusively of female patients, the potential influence of sex on postoperative outcomes could not be assessed. Consequently, the present results should be considered preliminary and require confirmation in larger, adequately powered prospective studies including more diverse patient populations.

## 5. Conclusions

BVFP paralysis could rarely present OSA symptoms. The present findings suggest that surgical treatment may improve sleep quality in patients with OSA symptoms associated with BVFP; however, given the limited sample size and the small number of controls, further studies are required to confirm these observations.

## Figures and Tables

**Table 1:** The values of the breathing parameters measured during sleep before and after operation. AHI—Apnea–Hypopnea Index; ODI—Oxygen Desaturation Index; BMI—Body Mass Index.

			Value
AHI	17.1 (6.65–61.75)	7.8 (4.2–15.0)	0.125
Snoring %	72.7 (47.8–73.85)	44.8 (11.7–81.95)	0.625
ODI	39.5 (6.6–58.5)	4.4 (1.5–12.1)	0.0625
Apnea	3 (1.45–34.7)	1.2 (0.5–7.7)	0.0625
Hypopnea	12 (5.15–28.15)	6.6 (2.35–7.3)	0.125
Mean SpO_2_	92.1 (91–95.3)	92.3 (91.05–95.6)	0.625
SpO_2_ < 90%	5.3 (0.1–19.2)	2.7 (0–9.4)	0.25
Mean SpO_2_ desaturation	4.1 (3.15–5.1)	3.7 (1.8–5.5)	0.625
Epworth Sleepiness Scale	5 (1–12.5)	3 (1–4.5)	0.25
Improvement of Sleep Quality after Surgery (yes or no)		Yes (n = 5)	
BMI	29.7 (21.7–32.9)		
Age	62 (54–64)		
Sex	Female (n = 5)		

## Data Availability

The data that support the findings of this study are available from the corresponding author upon reasonable request.

## Abbreviations

The following abbreviations are used in this manuscript:

BVFP	Bilateral Vocal Fold Paralysis
PSG	Polysomnography
ESS	Epworth Sleepiness Scale
RLNs	Recurrent Laryngeal Nerves
OSA	Obstructive Sleep Apnea
BMI	Body Mass Index
CPAP	Continuous Positive Airway Pressure
ODI	Oxygen Desaturation Index
AHI	Apnea–Hypopnea Index
